# Host Expression of the CD8 Treg/NK Cell Restriction Element Qa-1 is Dispensable for Transplant Tolerance

**DOI:** 10.1038/s41598-017-11780-2

**Published:** 2017-09-11

**Authors:** Blair T. Stocks, Christopher S. Wilson, Andrew F. Marshall, Lauren A. Brewer, Daniel J. Moore

**Affiliations:** 1 Department of Pathology, Microbiology, and Immunology, Vanderbilt University, Nashville, TN USA; 2 Department of Pediatrics, Ian Burr Division of Endocrinology and Diabetes, Vanderbilt University Medical Center, Nashville, TN USA

## Abstract

Disruption of the non-classical Major Histocompatibility Complex (MHC) Ib molecule Qa-1 impairs CD8 Treg and natural killer (NK) cell function and promotes a lupus-like autoimmune disease. This immune perturbation would be expected to enhance anti-transplant responses and impair tolerance induction, but the effect of Qa-1 deficiency on the transplant response has not been previously reported. Qa-1 deficiency enhanced CD4 TFH and germinal center (GC) B cell numbers in naïve mice and hastened islet allograft rejection. Despite enhanced immunity in B6.Qa-1^−/−^ mice, these mice did not generate an excessive primary CD4 TFH cell response nor an enhanced alloantibody reaction. Both CD8 Tregs and NK cells, which often regulate other cells through host Qa-1 expression, were targets of anti-CD45RB therapy that had not been previously recognized. However, B6.Qa-1^−/−^ mice remained susceptible to anti-CD45RB mediated suppression of the alloantibody response and transplant tolerance induction to mismatched islet allografts. Overall, despite enhanced immunity as demonstrated by augmented CD4 TFH/GC B cell numbers and hastened islet allograft rejection in naïve 12-week old Qa-1 deficient mice, the CD8 Treg/NK cell restriction element Qa-1 does not regulate the primary cellular or humoral alloresponse and is not required for long-term transplant tolerance.

## Introduction

We have recently established that lupus-prone B6.SLE123 mice are completely resistant to transplantation tolerance induction^1^. We demonstrated this resistance in a model of islet transplantation so that there was no pre-existing anti-graft autoimmunity. Despite the lack of pre-existing autoimmunity, the immune environment of B6.SLE123 mice defied reprogramming with tolerance-inducing therapy. We traced this failure to resistance of CD4 T cells to regulation by both CD4 and CD8 regulatory T cells (Tregs). CD8 Tregs have recently been established as potent regulators of islet allograft rejection^2^; however, it is not known how they are restricted to perform their function. An important class of regulatory CD8 T cells are restricted by the non-classical MHC class Ib protein Qa-1 (HLA-E)^3^. Deficiency in Qa-1 leads to a lupus-like disorder in mice and to enhanced T cell-dependent B cell responses^4^. The role of Qa-1 is unknown in islet transplantation.

Qa-1 interacts not only with a potent class of CD8 Tregs but also with NK cells^5^. CD8 Tregs bind Qa-1 via a TCR-restricted interaction whereas NK cells bind Qa-1 via their heterodimeric CD94/NKG2 complex. Although Qa-1-TCR ligation activates CD8 Tregs to lyse activated CD4 T Follicular Helper (TFH) cells via a perforin-dependent mechanism^4, 6, 7^, Qa-1-CD94/NKG2 ligation delivers an inhibitory signal to NK cells thereby preventing target CD4 T cell lysis^8, 9^. These reciprocal Qa-1 mediated interactions are essential in preventing autoimmune pathology.

In this report we explored the role of the CD8 Treg/NK cell restriction element Qa-1 during the transplant response. We determined that 12-week old, naïve Qa-1 deficient mice possessed enhanced CD4 TFH Cell and Germinal Center (GC) B Cell populations; however, absence of Qa-1 did not result in an unchecked expansion of CD4 TFH cells during primary alloimmunization, nor did it result in an excess production of alloantibody. Qa-1 deficient mice rejected islet allografts with faster kinetics than their Qa-1 sufficient counterparts, suggesting some enhancement in their baseline immune response. We further found that the tolerance inducing agent anti-CD45RB interacts with both CD8 Tregs and NK cells, many of which are Qa-1 restricted. However, Qa-1 deficient mice remained susceptible to anti-CD45RB mediated suppression of the alloantibody response and transplant tolerance induction to fully MHC-mismatched islet allografts. Overall, these data indicate that despite the role of Qa-1 in restraining autoimmunity and promoting CD8 Tregs and NK cell interactions, host expression of Qa-1 is dispensable during tolerance induction to allografted tissue suggesting that the islet-protective CD8 Tregs are not Qa-1 restricted.

## Materials and Methods

### Animals

The Institutional Animal Care and Use Committee (IACUC) at Vanderbilt University approved all procedures carried out during this study. All studies were carried out under this approved protocol in keeping with all relevant AAALAC guidelines and regulations. Mice were housed in a specific-pathogen free facility maintained by Vanderbilt University. All mice (C57BL6/J [B6], B6.129S6-H2-T23^tm1Cant^/J [B6.Qa-1^−/−^], C3H/HeJ [C3H]) were purchased from The Jackson Laboratory (Bar Harbor, ME). B6.Qa-1^−/−^ mice were originally developed by Harvey Cantor (Dana-Farber Cancer Institute, Boston, MA).

### Flow Cytometry

Fixed and permeabilized splenocytes were stained with fluorophore-conjugated antibodies purchased from either BD Biosciences (San Jose, CA) or eBioscience (San Diego, CA): B220(RA3-6B2), Bcl-6(K112-91), CD4(RM4), CD8α (53–6.7), CD45RB(C363.16A), CD45RB(C363.16 A), CD49b(DX5), CD122(TM-B1), Fas(Jo2), IgM(II/41), Ki67(B56), NK1.1(PK136), PD-1(J43). Qdm (AMAPRTLLL) and Preproinsulin II (ALWMRFLPL) peptides were synthesized by GenScript (Piscataway, NJ) and shipped to the NIH Tetramer Core (Emory University, Atlanta, GA) for folding into mouse Qa-1^b^ Tetramers labeled with APC. Samples were acquired on a BD LSRFortessa and analyzed by FlowJo (TreeStar, Ashland, OR).

### Alloimmunization and Alloantibody Titer Analysis

Thirty million splenocytes from Major Histocompatibility (MHC) mismatched C3H mice (H-2k) were intravenously (i.v.) injected into recipient B6 and B6.Qa-1^−/−^ mice (H-2b). For alloantibody studies, sera were isolated on days 0, 7, 14, 21, and 28 and incubated with target C3H splenocytes. Splenocytes were stained with antibodies directed at CD3ε(145-2C11) and IgG1(A85-1). The Median Fluorescence Intensity (MFI) of bound mouse IgG1 on target C3H CD3 T cells was used to assess relative alloantibody titer. For CD4 TFH cell enumeration studies, recipients were sacrificed and splenocytes analyzed by flow cytometry 7 days after alloimmunization.

### Islet Allografting and anti-CD45RB Therapy

Sub-capsular renal islet transplantation was carried out as previously described^10^. B6 and B6.Qa-1^−/−^ mice were made diabetic by streptozotocin (STZ, 200 mg/kg, Sigma Aldrich, St. Louis, MO) and transplanted with 400 allogeneic C3H islets. To induce transplant tolerance or study the effects of this agent, mice were intraperitoneally (i.p.) injected with 100 μg of the anti-CD45RB monoclonal antibody (BioXCell, West Lebanon, NH) on days 0, 1, 3, 5, and 7 after transplantation or initiation of the study^11^. Graft rejection was denoted via blood glucose readings above 250 mg/dL on two consecutive days. Animals achieving greater than 100 days of euglycemia were nephrectomized of their graft-containing kidney to confirm reliance on transplanted islet mass.

### Statistical Analysis

Statistical analysis was performed with GraphPad Prism V6 (La Jolla, CA), using two-tailed, non-parametric Mann-Whitney rank analysis for comparison of two non-normally distributed conditions. One-way or Two-way ANOVA followed by Tukey’s or Bonferroni’s multiple comparison’s post-test was used to compare multiple, normally distributed groups. Graft rejection was graphed as a Kaplan-Meier curve and compared by Log-rank statistical analysis. Data is presented as the mean with error bars denoting standard error of the mean (SEM). Statistical comparisons with p-values < 0.05 were deemed significant.

## Results

### Qa-1 deficiency slightly enhances CD4 TFH/GC B cell/CD8 Treg frequencies in aged 12-week old naive mice

**Figure 1:**
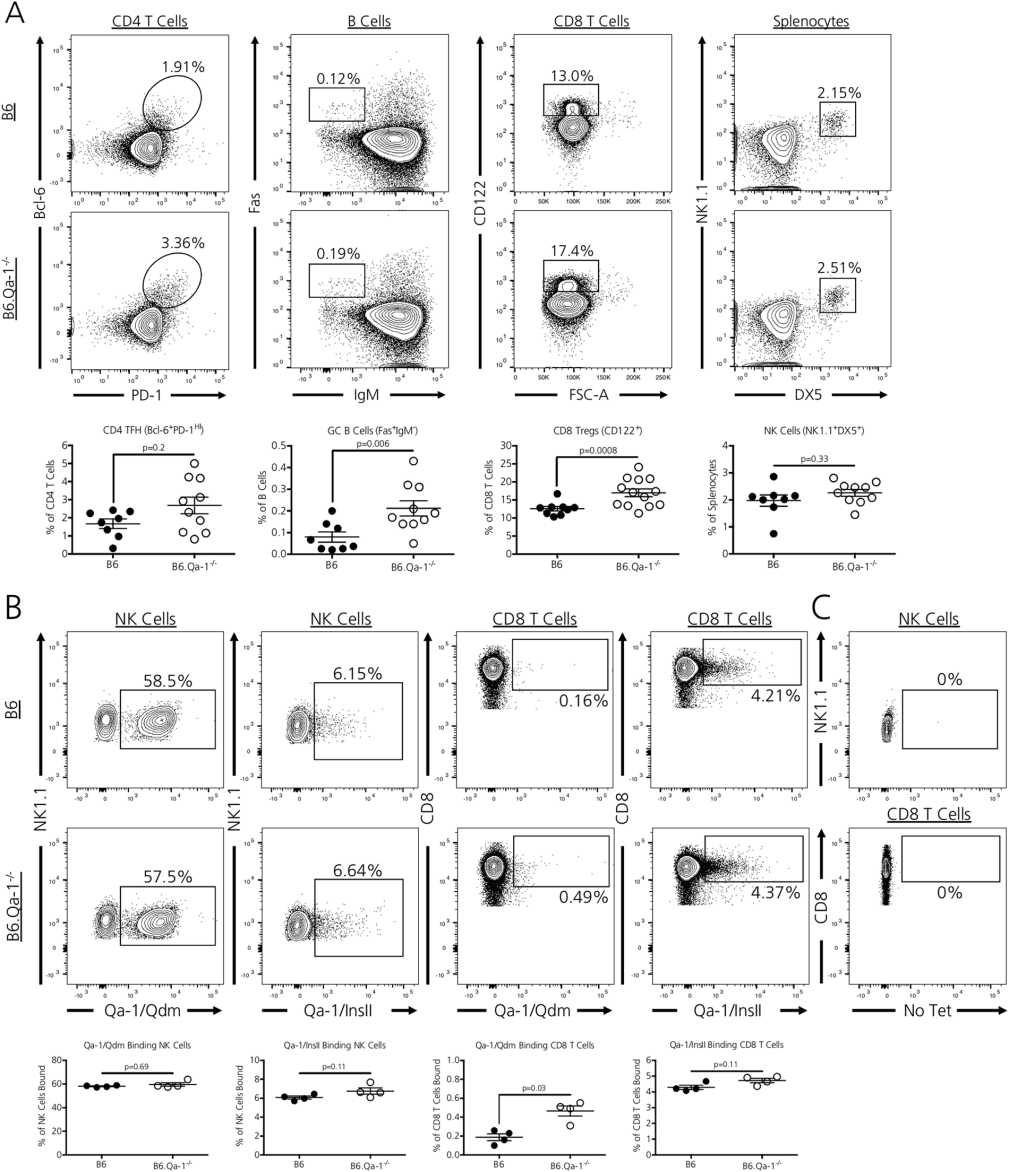
Qa-1 deficiency modestly alters peripheral immune cell proportions. (**A**) Naïve, 12-week old B6.Qa-1^−/−^ mice possess enhanced Germinal Center B Cell (B220 + IgM-Fas+) and CD8 T Regulatory Cell (CD8 + CD122+) splenic frequencies. There is a trend toward a slight increase in CD4 T Follicular Helper Cell (CD4 + PD-1 ^HI^Bcl-6+) highlighted in some animals. Overall splenic NK cell (DX5 + NK1.1+) frequencies remain unchanged. Data shown is pooled from three independent experiments (*n* = 8–13 mice per strain analyzed). (**B**) Naïve, 12-week old B6 and B6.Qa-1^−/−^ mice possess similar frequencies of Qa-1/Qdm and Qa-1/InsII tetramer-binding NK cells. Whereas B6 and B6.Qa-1^−/−^ mice possessed similar frequencies of Qa-1/InsII tetramer-binding CD8 T cells, B6.Qa-1^−/−^ mice posses approximately 3-fold higher frequencies of Qa-1/Qdm tetramer-binding CD8 T cells. Data shown is representative of two independent experiments (*n* = 4 mice per strain analyzed per experiment). (**C**) All gates for Qa-1 tetramer staining were set on samples not stained with Qa-1-APC labeled tetramers (Fluorescence Minus One). For all experiments, significance was determined by a two-tailed, non-parametric Mann-Whitney rank analysis. Calculated *p* values are shown.

The absence of Qa-1 does not alter T cell subpopulations in juvenile 4–6 week old mice^3^. However, as Qa-1 is required for the elimination of GC selective CD4 TFH cell populations^4^, we questioned whether 12-week old adult mice possessed a heighted CD4 TFH/GC B cell axis that may indicate heightened immunity due to inadequate, ongoing regulation by CD8 Tregs and/or NK cells. We assessed CD4 TFH cell and GC B cell populations along with suppressive CD8 Treg and NK cells in 12-week old, naive Qa-1 sufficient mice (B6) and Qa-1 null syngeneic counterparts (B6.Qa1^−/−^) [Fig. 1A]. Overall, B6.Qa-1^−/−^ mice demonstrated a non-significant trend towards expanded CD4 + PD-1 ^HI^Bcl-6 + TFH cells (p = 0.2) and had significantly expanded B220 + Fas + IgM- GC B cell (p = 0.006) splenic populations. Furthermore, B6.Qa-1^−/−^ mice possessed increased CD8 + CD122 + CD8 Treg splenic populations (p = 0.0008), a subset of CD8 T cells which includes Qa-1-restricted CD8 Tregs. The absence of Qa-1 did not alter splenic NK1.1 + DX5 + NK cell populations (p = 0.3).

### Qa-1 expression is not required for the selection of Qa-1 binding CD8 T/NK cells

We next questioned whether loss of Qa-1 expression altered the selection/development of Qa-1 binding CD8 T cell and NK cell populations; selective loss of these Qa-1 binding populations could result in an enhanced transplant response due to inadequate CD4 TFH cell pruning. We assessed Qa-1 binding within CD8 T and NK cell populations using Qa-1 tetramers loaded with either the Qa-1 predominant modifier (Qdm) peptide^12^ or a known Qa-1 binding peptide from the Preproinsulin II (InsII)^13^. In line with previous data, approximately half of NK1.1 + DX5 + NK cells bound the Qa-1/Qdm complex in Qa-1 sufficient B6 mice^14^. Strikingly, the genetic absence of Qa-1 did not result in a selective loss of these Qa-1/Qdm binding NK cells; we observed that a similar percentage of NK cells in B6.Qa-1^−/−^ mice remained capable of binding Qa-1/Qdm tetramers [Fig. 1B, first column (p = 0.69)]. This observation is likely due to the fact that NK cells from both strains expressed similar levels of the Qa-1 interacting heterodimer CD94/NKG2A (data not shown), the expression of which may not rely on the presence of Qa-1. Interestingly, we observed a slight expansion of Qa-1/Qdm binding CD8 T cells in B6.Qa-1^−/−^ mice [Fig. 1B, third column (p = 0.03)], thereby suggesting that Qa-1 expression is dispensable for the selection of Qa-1 binding CD8 T cells. This hypothesis is further supported by our finding that Qa-1 deficient mice possessed similar percentages of Qa-1/InsII binding CD8 T and NK cell populations as compared to their Qa-1 sufficient counterparts [Fig. 1B, second (p = 0.11) and fourth (p = 0.11) columns]. To ensure accurate Qa-1 tetramer staining within samples, all gates were set on CD8 T Cell/NK cell samples stained without tetramers [Fig. 1C, Fluorescence Minus One for Qa-1- APC tetramers].

### Qa-1 deficiency does not result in an unchecked primary CD4 TFH cellular alloresponse nor does it enhance humoral alloantibody production

**Figure 2:**
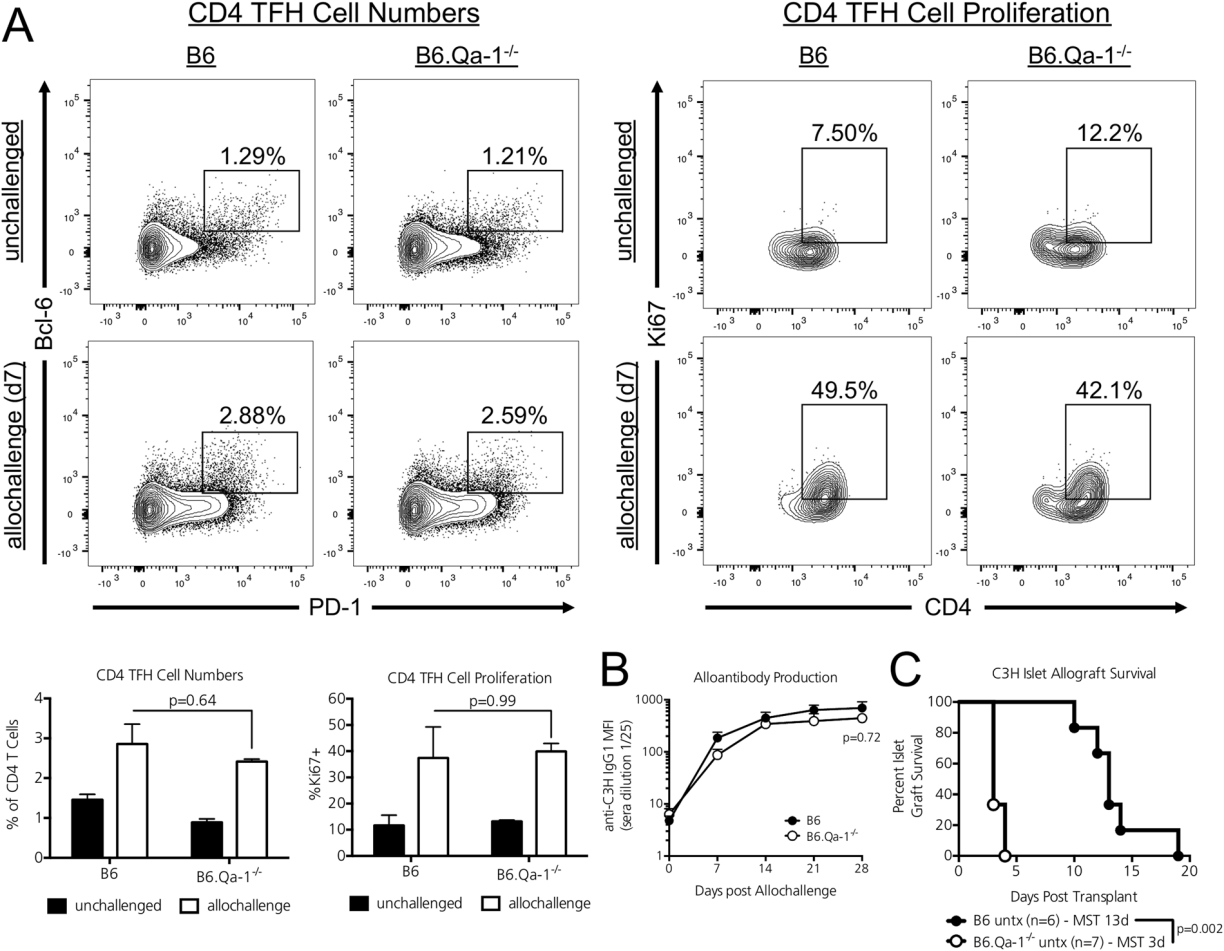
Qa-1 deficiency hastens islet allograft rejection but does not significantly alter the germinal center alloresponse. (**A**) 12-week old B6 and B6.Qa-1^−/−^ mice (H2-b) were i.v. injected with 30 million fully-MHC mismatched C3H splenocytes (H2-k) or left untreated and sacrificed 7 days later. Splenic CD4 TFH cell frequencies (left panel) and proliferation (right panel, Ki-67 positivity) in allochallenged mice did not differ between Qa-1 sufficient or deficient recipients. *n* = 3 mice per strain/condition analyzed; representative of 3 independent experiments. Significance was determined by two-way ANOVA followed by Tukey’s multiple comparison’s post-test for normally distributed data. Adjusted *p* values are shown for groups of interest. (**B**) In a second cohort of recipients, 12-week old B6 and B6.Qa-1^−/−^ mice (H2-b) were i.v. injected with fully-MHC mismatched C3H splenocytes (H2-k), bled weekly, and anti-C3H IgG1 alloantibody titers analyzed by flow cytometry using target C3H cells. Overall, the absence of Qa-1 did not result in augmented anti-C3H IgG1 alloantibody titers. *n* = 4–5 mice per strain analyzed, combined from two independent experiments. Significance determined by two-way ANOVA, followed by Bonferroni’s post-test. An adjusted *p* value is shown for Day 28. (**C**) STZ treated, diabetic B6 and B6.Qa-1^−/−^ recipients were transplanted with fully MHC-mismatched C3H islet allografts and left untreated. Overall, the absence of Qa-1 significantly hastened C3H islet allograft rejection (B6.Qa-1^−/−^ MST – 3d; B6 MST – 13d). Significance determined by Log-Rank test. *p* values are shown on the graph and the number of graft recipients is denoted by *n* on the graph.

Because 12-week old naïve B6.Qa-1^−/−^ mice possessed enhanced CD4 TFH/GC B Cell splenic populations, we questioned whether this heightened immunity would result in an enhanced cellular alloresponse. B6 and B6.Qa-1^−/−^ mice (H2-b) were i.v. injected with 30 million fully MHC-mismatched C3H splenocytes (H-2k). Seven days later splenic CD4 TFH, GC B cell, and CD8 Tregs populations were assessed. Although we observed no significant changes in CD8 Treg or GC B cell populations in naïve vs. allochallenged recipients at this early time point (data not shown), both strains mounted a CD4 TFH cellular alloresponse. The percentage of CD4 TFH cells [Fig. 2A, left columns, p = 0.64] and their proliferation as assessed by Ki-67 positivity [Fig. 2A, right columns, p = 0.99] did not differ between Qa-1 sufficient or deficient recipients during allochallenge.

Although the absence of Qa-1 did not result in an enhanced CD4 TFH cellular alloresponse, it remained possible that the lack of this CD8 Treg/NK cell restriction element could result in an unchecked humoral alloresponse. Thus, a second cohort of B6 and B6.Qa-1^−/−^ mice (H2-b) were i.v. injected with 30 million fully MHC-mismatched C3H splenocytes (H-2k). Mice were bled on days 7, 14, 21, and 28 after allochallenge and the production of anti-C3H IgG1 alloantibody assessed by flow cytometry using target C3H splenocytes incubated with recipient serum. Overall, the absence of Qa-1 did not result in an unchecked humoral alloresponse; both strains generated similar anti-C3H IgG1 alloantibody titers [Fig. 2B, p = 0.72 at Day 28].

### Qa-1 deficiency hastens primary islet allograft rejection

It remained possible that the heightened immunity characteristic of Qa-1 deficient mice may render these recipients less susceptible to allograft engraftment. Compared to WT B6 mice, Qa-1 deficient animals exhibit enhanced CD4 T cell proliferation and Interferon gamma (IFNg) secretion within draining lymph nodes during infectious challenge^3^. As IFNg is a potent mediator of islet allograft rejection^15^, we assessed whether Qa-1 deficiency alone alters primary islet allograft rejection kinetics. STZ-treated, diabetic B6 and B6.Qa-1^−/−^ mice were transplanted with C3H islet allografts and left untreated. Whereas islet allograft Median Survival Time (MST) in Qa-1 sufficient B6 mice was 13d as previously reported, B6.Qa-1^−/−^ recipients rejected their islet allografts with a MST of 3d [Fig. 2C, p = 0.002 by log-rank analysis].

### The tolerance inducing agent anti-CD45RB targets CD8 Tregs and NK cells

**Figure 3:**
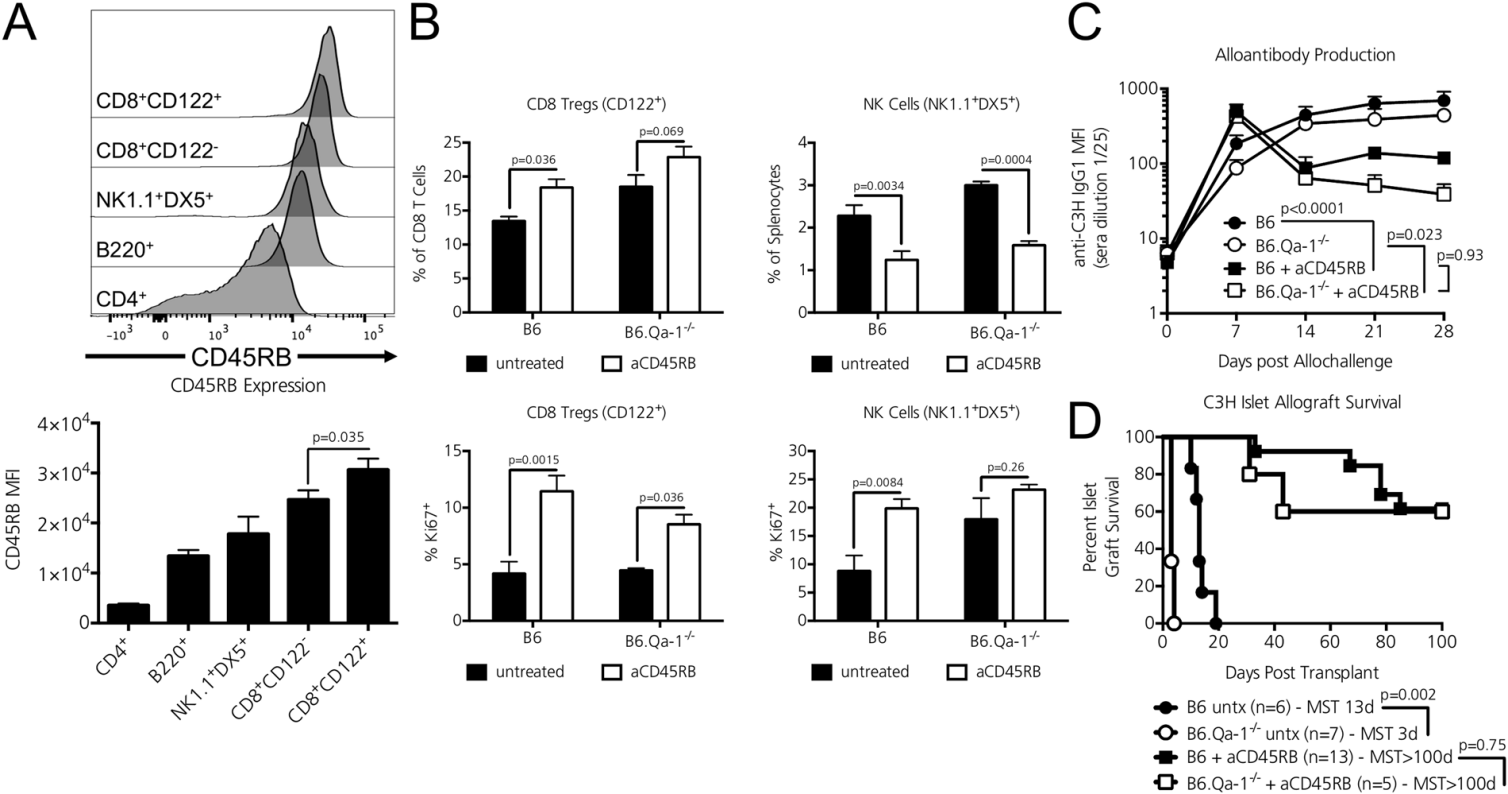
Anti-CD45RB interacts with Qa-1 restricted cells but Qa-1 is dispensable for tolerance induction. (**A**) Expression of the CD45RB isoform on gated CD4+ T cells, B220+ B cells, NK1.1 + DX5 + NK cells, naïve CD122- CD8 T cells, and CD122 + CD8 Tregs was assessed by flow cytometry. Significance determined by One-way ANOVA for normally distributed data, followed by Tukey’s post-test for multiple comparisons. A significant *p* value is shown on the graph for two groups of interest. *n* = 3 mice per strain analyzed. (**B**) 12-week old B6 and B6.Qa-1^−/−^ mice were left untreated or received a standard 7-day course of anti-CD45RB. CD8 Treg and NK cell populations were assessed on day 8. Overall, anti-CD45RB significantly increased CD8 Treg frequencies and proliferation (as measured by Ki-67 positivity) in both strains. Although this agent significantly reduced splenic NK cell frequencies in both strains, anti-CD45RB significantly increased NK cell proliferation in B6 mice. Data shown is pooled from two independent experiments (*n* = 3–7 mice per strain analyzed). Significance determined by two-way ANOVA, followed by Bonferroni’s post-test. Adjusted *p* values of interest are shown on the graph. (**C**) 12-week old B6 and B6.Qa-1^−/−^ mice (H2-b) were i.v. injected with 30 million fully-MHC mismatched C3H splenocytes (H2-k) and left untreated or received 100 ug i.p. injections of the tolerance inducing agent anti-CD45RB on days 0,1,3,5,7 relative to allochallenge (d0). Mice were bled weekly and anti-C3H IgG1 alloantibody titers analyzed by flow cytometry using target C3H cells. Overall, anti-CD45RB therapy significantly reduced anti-C3H IgG1 alloantibody production in both B6 and B6.Qa-1^−/−^ recipients vs. their untreated counterparts. There was no significant difference in anti-C3H IgG1 alloantibody titers between anti-CD45RB treated B6 and B6.Qa-1^−/−^ recipients. *n* = 4–5 mice per strain analyzed, pooled from two independent experiments. Significance determined by two-way ANOVA, followed by Tukey’s post-test. Adjusted *p* values of interest comparing Day 28 are shown on the graph. (**D**) STZ treated, diabetic B6 and B6.Qa-1^−/−^ recipients were transplanted with fully MHC-mismatched C3H islet allografts and administered 100ug i.p. injections of the tolerance inducing agent anti-CD45RB on days 0, 1, 3, 5, 7 relative to the day of transplantation (d0). Overall, the absence of Qa-1 did alter the susceptibility to long-term transplant tolerance induction. Animals achieving 100 days of tolerance were nephrectomized of their allograft-containing kidneys. All animals returned to hyperglycemia within 2 days of surgery confirming graft function for maintenance of euglycemia (not shown). Significance determined by Log-Rank test. *p* values are shown on graph and the number of graft recipients is denoted by *n* on the graph.

To examine the role of Qa-1 restricted cell types during tolerance induction, we first determined whether CD8 Tregs and NK cell populations were targets of the commonly used tolerogenic therapy anti-CD45RB^16^. In comparison to CD4 T cells that are known targets of anti-CD45RB^17^, there exist little data describing the tolerogenic effects of anti-CD45RB on CD8 T cell and NK cell populations. To date, it has only been reported that anti-CD45RB dampens CD8 T cell islet allograft infiltrate^18^ and that NK cells are required for anti-CD45RB mediated tolerance induction^19^. Thus, we sought to clarify the role of CD8 Tregs and NK cells during anti-CD45RB therapy. We found that both NK1.1 + DX5 + NK cells and CD8 + CD122 + CD8 Tregs expressed high levels of the CD45RB isoform in naïve B6 splenocytes [Fig. 3A]. B6 and B6.Qa1^−/−^ mice were treated with a standard 7-day course of anti-CD45RB therapy or left untreated^11^. Overall, anti-CD45RB increased splenic CD8 Treg percentages and proliferation in both strains [Fig. 3B, left column]. Although anti-CD45RB reduced overall NK cell splenic frequencies in both strains (as previously observed in B6 mice^19^), it significantly increased NK cell proliferation in B6 recipients. [Fig. 3B, right column]. All flow plots are shown in Supplementary Figure 1.

### Qa-1 deficient recipients remain susceptible to anti-CD45RB mediated alloantibody suppression and transplant tolerance induction

The tolerogenic agent anti-CD45RB not only induces antigen-specific allograft tolerance, but it also efficiently dampens the humoral alloresponse^20^, which could relate to a role for NK cells and CD8 Tregs. Thus, we assessed whether the CD8 Treg/NK cell restriction element Qa-1 was required during anti-CD45RB mediated suppression of the humoral alloresponse. In line with previous reports^20^, a seven-day course of anti-CD45RB effectively dampened the anti-C3H IgG1 alloantibody response in Qa-1 sufficient B6 recipients [Fig. 3C, anti-CD45RB (black boxes) vs. untreated (black circles), p < 0.0001]. Interestingly, anti-CD45RB similarly dampened the humoral alloantibody response in treated Qa-1 deficient mice [Fig. 3C, anti-CD45RB (white boxes) vs. untreated (white circles), p = 0.023]. Overall, we observed no significant difference between anti-C3H IgG1 alloantibody titers between anti-CD45RB treated B6 and B6.Qa-1^−/−^ mice [Fig. 3C, B6 + anti-CD45RB (black boxes) vs. B6.Qa-1^−/−^ + anti-CD45RB (white boxes), p = 0.93].

Finally, we questioned whether the CD8 Treg/NK cell restriction element Qa-1 was required for long-term transplant tolerance induction mediated by anti-CD45RB. STZ-treated, diabetic B6 and B6.Qa-1^−/−^ mice were transplanted with C3H islet allografts and administered a seven-day course of anti-CD45RB. Overall, 60% of B6.Qa-1^−/−^ mice achieved long-term tolerance to their islet allografts, a percentage that did not differ significantly from their Qa-1 sufficient B6 counterparts [Fig. 3D, p = 0.75]. To confirm allograft tolerance and reliance on the transplanted islet graft for maintenance of euglycemia vs. endogenous islet recovery, all recipients demonstrating >100 days of euglycemia were nephrectomized of their kidney containing allografts. All recipients returned to hyperglycemia within two days, thereby confirming intact graft function and tolerance to the MHC-mismatched islet allograft (data not shown).

## Discussion

Qa-1 restricted interactions limit the degree of the immune response during both natural immunization and the development of autoimmunity^3, 4^. Critical Qa-1 restricted cells include CD8 Tregs and NK cells, both of which are important during tolerance induction. Matching at the Qa-1 (HLA-E) locus has been found to diminish GVHD in bone marrow transplant^21^, but its role in other cell and organ transplants had not been determined. We anticipated that the alloresponse would be similarly enhanced by deficiency in Qa-1 and that this enhancement would lead to diminished tolerance induction, as is seen in type 1 diabetes and lupus where Qa-1 mediated regulation plays a protective role^1, 4, 11, 22^. Because of the recent identification of a potent role for CD8 Tregs in islet allograft protection^2^ and the long literature identifying Qa-1 restricted CD8 Tregs^23, 24^, we investigated whether there is a requirement for Qa-1 in islet allograft protection. In our assessment of the transplant response, we did not identify any increased allo-immune response at the T or B cell level despite observing that Qa-1 deficiency hastened islet allograft rejection in the absence of tolerance inducing therapy. We were able to determine that tolerance-inducing therapy anti-CD45RB had a positive effect on CD8Treg and NK cell activation. However, the capacity of these cells to interact with host target cells via Qa-1 mediated interactions was not needed for anti-CD45RB mediated tolerance to a fully mismatched islet transplant.

These findings contribute to our understanding of the role of CD8 Tregs and NK cells in the prevention of allograft rejection. It was recently reported that a subpopulation of CD8 T cells positive for the IL-2 Receptor Beta chain (CD122) delay islet and skin allograft rejection in an IL-10 dependent manner and may be more potent in this regard than CD4 + Tregs, but it was not known how they were restricted leading us to consider a potential role for Qa-1^2, 25, 26^. Moreover, it is also known that perforin-secreting NK1.1 + NK cells are required during anti-CD40L and anti-LFA-1 mediated transplant tolerance induction to islet allografts^27^. The restriction element Qa-1, with which both some CD8 Tregs and NK cells interact, is essential for adequate suppression of activated CD4 T cells in other models of immunity^8^. Whether host Qa-1 expression is similarly required for adequate CD8 Treg/NK cell mediated suppression of the alloresponse had not been investigated. Overall, we determined that the absence of host Qa-1 does not result in an enhanced primary cellular or humoral alloresponse, nor does it affect the ability of the tolerogenic agent anti-CD45RB to dampen the humoral alloresponse or imbue long-term allograft tolerance, suggesting that the allo-regulatory CD8 and NK cell activities are not Qa-1 restricted or that other regulatory cell types can readily make up for any potential contribution from this interaction with this non-classical MHC during transplantation.

Unlike Foxp3 + CD4 Tregs that are believed to suppress the initiation of the primary effector T cell response, suppression mediated by CD8 Tregs may play a more predominant role during the secondary immune response; evidence for this model was first documented by Hu *et al*. who found that Qa-1 sufficient B6 mice were protected from subsequent experimental autoimmune encephalitis (EAE) after secondary immunization^3^. In contrast, Qa-1 deficient B6 mice readily succumbed to EAE in this model. Accordingly, it could be speculated that CD8 Tregs may be necessary in settings where there is prior sensitization against the graft (mimicking the clinical scenario of retransplantation). In fact, recent work by Long and colleagues demonstrated that CD8 Tregs previously exposed to graft antigen delayed cardiac allograft rejection when transferred alongside allograft-sensitized memory CD4 T cells. Such protection was mitigated when recipients received concomitant injections of an anti-Qa-1 blocking antibody^28^. Thus, these data support a role for CD8-Treg/Qa-1 mediated protection of allografts during the secondary alloresponse, while we have focused on tolerance induction during primary engraftment where we find Qa-1 to be dispensable.

Although we observed that the absence of host expression of Qa-1 did not alter the ability of NK cell populations to bind Qa-1/Qdm or Qa-1/InsII tetramers (most likely due to intact CD94/NKG2 expression), we found it surprising that a significant portion of CD8 T cells retained the ability to bind Qa-1/Qdm and Qa-1/InsII tetramers despite previous reports demonstrating that adequate Qa-1 expression was required for the selection of Qa-1/TCR restricted CD8 T cells^29^. Thus, it could be inferred from our results that despite the absence of host cell expression of Qa-1, a subset of CD8 T cells retain an ability to bind Qa-1, potentially through non-TCR mediated interactions (e.g. CD94/NKG2 as a subset of murine CD8 T cell express these receptors^30^).

Overall, we determined that the absence of host cell expression of the non-classical MHC Class Ib molecule Qa-1 hastens islet allograft rejection in the absence of tolerance-inducing therapy. However, Qa-1 deficiency did not augment the primary cellular or humoral alloimmune response, nor did it affect susceptibility to anti-CD45RB mediated transplant tolerance induction suggesting that Qa-1 interactions are not an absolute requirement in the establishment of transplantation tolerance. It is likely that other targets of anti-CD45RB, such as CD4 Tregs that express intermediate levels of CD45RB, are sufficient for tolerance induction to the primary transplant. Our results do not rule of the possibility of a non-Qa-1 restricted CD8 Treg/NK cell populations in attenuating the transplant response, especially in light of our data demonstrating that CD122 + CD8 Tregs and NK cells are targets of the tolerogenic therapy anti-CD45RB, or that Qa-1 restricted cells may be important in re-transplantation. Having determined that Qa-1 is not required for the primary induction of stable immune regulation, future studies remain highly warranted to determine how graft-protective CD8 Tregs recognize their targets and execute their function during the alloresponse in order to capture their potential to enhance transplantation.

## Electronic supplementary material

Supplementary Figure
