# Supplementary material for: Host Expression of the CD8 Treg/NK Cell Restriction Element Qa-1 is Dispensable for Transplant Tolerance
Source: Sci Rep. 2017 Sep 11;7:11181. doi: 10.1038/s41598-017-11780-2 (PMC5593978; doi:10.1038/s41598-017-11780-2)
Supplement: Supplementary file 1 — Supplementary Figure [file 41598_2017_11780_MOESM1_ESM.pdf]

Host Expression of the CD8 Treg / NK Cell Restriction Element Qa-1 is Dispensable for Transplant Tolerance

Blair T. Stocks, PhD[1], Christopher S. Wilson, BS[1], Andrew F. Marshall, BA[2], Lauren A. Brewer[2], and Daniel J. Moore, MD, PhD[1,2]

Supplementary Figure 1. Flow plots of data graphed in **Figure 3B**. Briefly, 12-week old B6 and B6.Qa-1$^{-/-}$ mice were left untreated or received a standard 7-day course of anti-CD45RB. Splenic CD8 Treg and NK Cell population frequencies and proliferation were assessed on day 8.

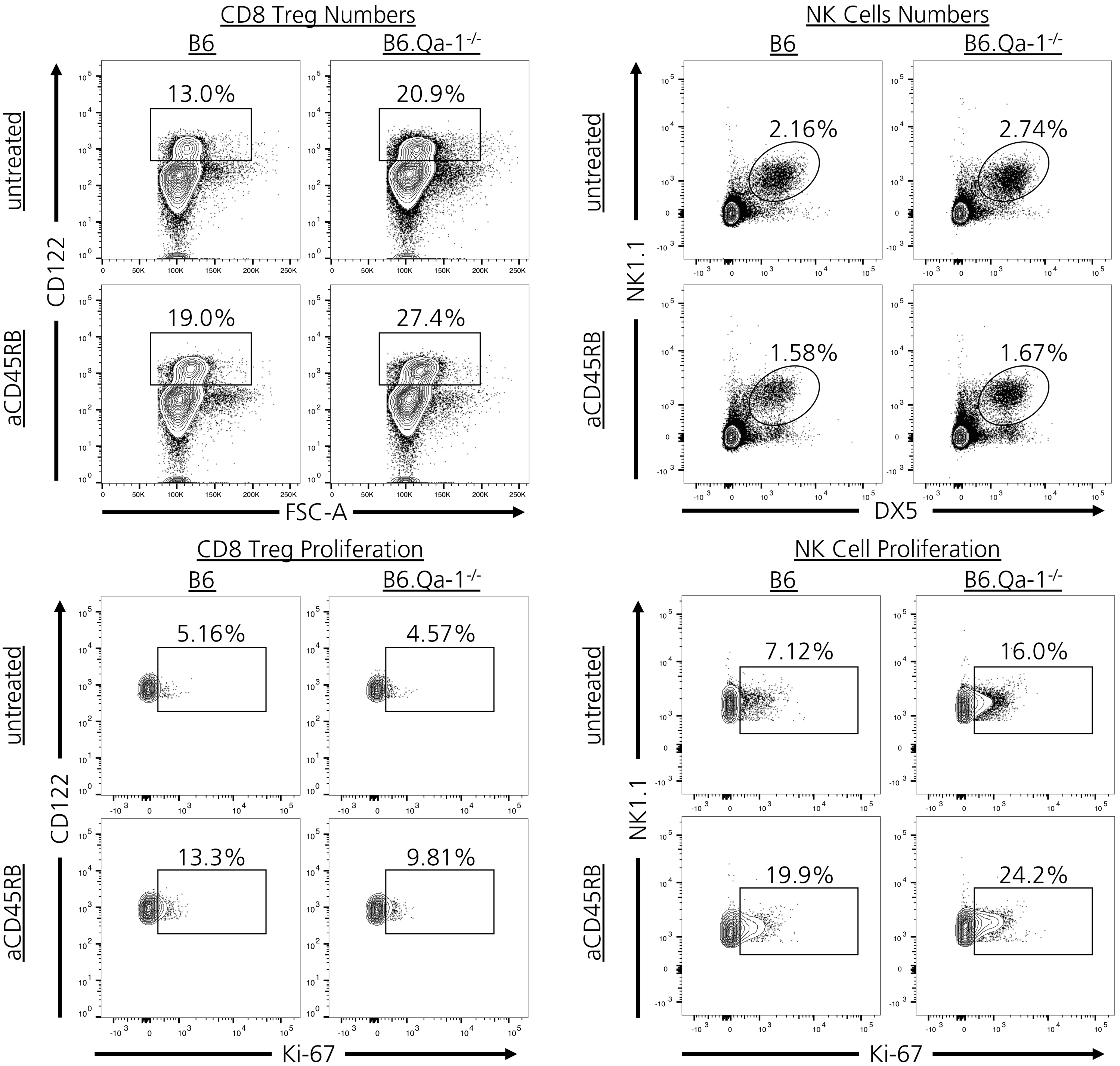
CD8 Treg Numbers
B6
B6.Qa-1-/-
untreated
aCD45RB
13.0%
20.9%
19.0%
27.4%
CD122
FSC-A
NK Cells Numbers
B6
B6.Qa-1-/-
untreated
aCD45RB
2.16%
2.74%
1.58%
1.67%
NK1.1
DX5
CD8 Treg Proliferation
B6
B6.Qa-1-/-
untreated
aCD45RB
5.16%
4.57%
13.3%
9.81%
CD122
Ki-67
NK Cell Proliferation
B6
B6.Qa-1-/-
untreated
aCD45RB
7.12%
16.0%
19.9%
24.2%
NK1.1
Ki-67
